# Pathological and Perioperative Outcomes of Conversion Hepatectomy After Contemporary Combination Downstaging for Initially Unresectable Hepatocellular Carcinoma: A Systematic Review

**DOI:** 10.3390/curroncol33080453

**Published:** 2026-07-28

**Authors:** Codruta Craciun, Livia Stanga, Danut Dejeu, Ana-Maria Davidoiu, Adrian Cosmin Ilie, Patricia Octavia Mazilu, Lavinia Craciun, Stelian Pantea

**Affiliations:** 1 Doctoral School, “Victor Babes” University of Medicine and Pharmacy Timisoara, 300041 Timisoara, Romania; codruta.craciun@umft.ro; 2 Discipline of Microbiology, “Victor Babes” University of Medicine and Pharmacy Timisoara, 300041 Timisoara, Romania; stanga.livia@umft.ro; 3 Surgical Oncology Department, Emergency County Hospital Oradea, 410169 Oradea, Romania; 4 Department of Functional Sciences, Discipline of Public Health, Center for Translational Research and Systems Medicine, “Victor Babes” University of Medicine and Pharmacy Timisoara, 300041 Timisoara, Romania; ilie.adrian@umft.ro; 5 Faculty of Medicine, “Victor Babes” University of Medicine and Pharmacy Timisoara, Eftimie Murgu Square 2, 300041 Timisoara, Romania; patrimazilu@yahoo.com; 6 Department of Anatomy and Embryology, “Victor Babes” University of Medicine and Pharmacy Timisoara, 300041 Timisoara, Romania; craciun.lavinia@umft.ro; 7 Department X, Surgical Emergencies Clinic, “Victor Babes” University of Medicine and Pharmacy Timisoara, 300041 Timisoara, Romania; pantea.stelian@umft.ro

**Keywords:** hepatocellular carcinoma, liver resection, conversion therapy, downstaging, hepatectomy, salvage surgery, immunotherapy, tyrosine kinase inhibitor, pathological complete response, perioperative outcomes

## Abstract

**Background and Objectives:** Conversion therapy has expanded treatment options for patients with initially unresectable hepatocellular carcinoma (HCC), but the surgical literature remains focused more often on radiologic response than on the pathological, perioperative, and postoperative outcomes of patients who actually proceed to hepatectomy. This focused systematic review aimed to synthesize the available evidence on conversion hepatectomy after contemporary combination downstaging for initially unresectable HCC. **Materials and Methods:** A structured PubMed/MEDLINE search with backward reference-list screening was performed and last updated on 3 February 2026. The full Boolean strategy, field tags, and eligibility framework are now reported explicitly. Because the literature was observational and clinically heterogeneous, findings were synthesized narratively and complemented by structured assessments of reporting completeness, potential cohort overlap, and study-level bias. **Results:** Fourteen studies were included, nearly all retrospective and predominantly from East Asia. Treatment platforms clustered into systemic doublets, systemic plus HAIC strategies, and broader locoregional–systemic triplet or multimodal approaches. Across studies reporting pathological response, pathological complete response ranged from 28.0% to 50.0%, while R0 resection ranged from 85.7% to 100%, where stated. Postoperative morbidity ranged from 14.3% to 71.4%, and major complication rates from 9.5% to 16.9%; however, extent of resection, liver reserve, post-hepatectomy liver failure, transfusion, and perioperative mortality were not uniformly reported. Most studies carried moderate-to-high overall concerns for bias because of response-based surgical selection, heterogeneous denominators, incomplete perioperative reporting, and possible partial overlap among some cohorts. **Conclusions:** The available literature suggests that conversion hepatectomy can be feasible and oncologically meaningful in carefully selected patients treated in experienced centers, but current evidence remains hypothesis-generating rather than practice-standardizing because it is observational, heterogeneous, and incompletely reported.

## Simple Summary

For some patients with advanced hepatocellular carcinoma, modern drug combinations and locoregional therapies can shrink or biologically control the tumor enough to make liver surgery possible. This review examined what happens after those patients actually reach hepatectomy. Across the available studies, surgeons often achieved complete tumor removal, and a meaningful proportion of patients had no viable tumor left in the resected specimen. However, the evidence comes mostly from retrospective, highly selected cohorts treated in experienced East Asian centers, and important surgical details were not reported consistently. These findings suggest that conversion hepatectomy is promising for carefully chosen patients, but stronger prospective studies with standardized reporting are still needed before the approach can be broadly standardized.

## 1. Introduction

Hepatocellular carcinoma (HCC) remains one of the leading causes of cancer-related death worldwide and continues to impose a substantial surgical and public-health burden despite advances in surveillance, imaging, and systemic therapy [1,2,3,4,5,6]. A large proportion of patients still present with multifocal disease, macrovascular invasion, an insufficient future liver remnant, impaired liver function, or anatomically unfavorable lesions that preclude immediate curative resection. In parallel, the modern therapeutic landscape has changed rapidly. First-line immunotherapy-based combinations, tyrosine kinase inhibitors, and intensified locoregional regimens have improved radiologic response rates compared with older systemic monotherapy paradigms, thereby reopening the question of whether selected patients with initially unresectable tumors can be converted to surgery [3,5,7,8,9]. This evolution is particularly relevant to liver surgery because resection remains one of the few potentially curative modalities for HCC outside transplantation. Consequently, surgeons increasingly encounter patients whose disease biology appears to improve under treatment, yet whose operative candidacy after downstaging is not fully defined. In this setting, the decision to proceed to hepatectomy depends not only on tumor shrinkage but also on evolving concepts of technical resectability, oncologic benefit, liver reserve, and the quality of pathological response achieved before surgery [2,3,4,5,6].

The concept of conversion therapy in liver oncology differs from classic palliative disease control. Its goal is to use preoperative treatment to transform a previously unresectable cancer into a lesion or disease pattern amenable to complete resection while preserving sufficient functional liver parenchyma [10,11,12,13]. In HCC, conversion strategies may involve targeted therapy plus immune checkpoint blockade, transarterial chemoembolization (TACE), hepatic arterial infusion chemotherapy (HAIC), radiation-based approaches, or multimodal combinations tailored to portal vein tumor thrombus, tumor burden, or underlying cirrhosis [5,10,11,12,13,14]. This framework has attracted growing attention because combination therapy can induce substantial necrosis and vascular response even in advanced-stage tumors, potentially shifting management from chronic disease suppression toward curative-intent local therapy. However, conversion therapy also introduces new uncertainties for liver surgeons. Radiologic response does not always equate to pathological sterilization; tissue planes may be altered by treatment-related inflammation or fibrosis, and liver function may deteriorate despite apparent tumor control. Therefore, any meaningful synthesis of this field must examine the postoperative population directly, rather than extrapolating from response rates in unresectable non-surgical cohorts [11,12,13,14,15].

Recent narrative reviews and broad meta-analyses indicate that conversion therapy for unresectable HCC is an expanding field, but they often pool highly heterogeneous studies and focus primarily on response rates, conversion rates, or overall treatment efficacy rather than on the detailed operative and pathological outcomes of the patients who actually undergo liver resection [11,12,13,14,15]. A targeted scoping check performed during the present review process did not identify a dedicated PubMed-indexed systematic review focused specifically on pathological and perioperative outcomes after conversion hepatectomy in the modern combination-therapy era; instead, the existing review literature addressed broader conversion-therapy questions, triple-therapy salvage surgery in selected Chinese cohorts, or mixed comparisons of surgery versus non-surgery after response [11,12,13,14,15]. This distinction matters because the surgical endpoint is biologically richer than the radiologic endpoint. Once resection is achieved, investigators can evaluate margin status, pathological complete response, residual viable tumor, microvascular invasion, treatment-related fibrosis, postoperative liver failure, recurrence-free survival, and the clinical relevance of adjuvant decisions. These are the endpoints most likely to inform multidisciplinary boards deciding whether radiologic responders should move forward to hepatectomy rather than continue medical therapy alone.

A further reason to focus on conversion hepatectomy is that modern HCC management is increasingly biomarker- and trajectory-driven rather than purely stage-based [2,5,10,14,15]. Tumor size reduction remains important, but contemporary surgical decision-making also considers alpha-fetoprotein kinetics, disappearance or persistence of vascular tumor thrombus, expected future liver remnant, portal hypertension, contralateral liver quality, and the depth of treatment response across all intrahepatic and extrahepatic sites. Surgeons must also interpret whether a complete radiologic response truly represents eradication of viable cancer or merely an imaging surrogate masking residual microscopic disease. Pathology from the resected specimen therefore becomes a critical source of truth. The growing use of pathological complete response, major pathological response, and tumor regression grade in the post-conversion setting reflects a broader shift toward integrating biological response into operative strategy, similar to other gastrointestinal malignancies. Yet the reporting of these endpoints in liver-surgery studies remains inconsistent, and their relationship to recurrence is still being defined [10,11,12,13,14,15].

From a practical standpoint, the perioperative dimension is equally important. Patients who undergo hepatectomy after conversion therapy are not typical primary-resection candidates. Many have received prolonged systemic treatment, repeated arterial interventions, or combined modality exposure that may alter parenchymal quality, biliary planes, coagulation, wound healing, and vascular handling. These factors can influence operative duration, blood loss, need for transfusion, postoperative complications, and the risk of post-hepatectomy liver failure even when the tumor becomes technically removable. At the same time, early reports suggest that when surgery is feasible, short-term outcomes may remain acceptable, and long-term survival may compare favorably with continued non-surgical treatment in selected responders [3,5,11,12,13,14,15]. The challenge is separating true surgical opportunity from selection bias. A descriptive synthesis centered on the resected cohorts can help clarify which operative outcomes have already been observed in practice, which pathological endpoints recur most consistently, and where the evidence remains too immature for firm clinical standardization.

Accordingly, the aim of this systematic review was to synthesize the available PubMed-indexed evidence on conversion hepatectomy after contemporary combination downstaging for initially unresectable HCC. The review was intentionally focused on the postoperative population and on variables of greatest relevance to hepatobiliary surgeons and multidisciplinary HCC teams: study design and treatment platforms, timing of surgery, perioperative safety, margin status, pathological response, recurrence patterns, survival outcomes, and prognostic factors derived from resected specimens. By separating this focused question from the broader literature on conversion therapy efficacy, the present manuscript seeks to provide a more clinically useful map of what is currently known about the patients who successfully cross the threshold from unresectable disease to liver resection. It also identifies the methodological limitations that currently prevent a definitive pooled estimate, thereby helping frame future prospective studies and review projects in this rapidly evolving area of liver surgery [11,12,13,14,15].

## 2. Materials and Methods

This study was designed as a focused systematic review of PubMed/MEDLINE-indexed literature evaluating conversion hepatectomy for initially unresectable hepatocellular carcinoma after contemporary combination downstaging. The revised manuscript was structured to align more completely with PRISMA 2020 reporting principles, and the decision not to perform meta-analysis remained prespecified because the evidence base was clinically and methodologically heterogeneous (Table S1) [16,17]. The clinical question was intentionally narrower than a broad review of conversion therapy efficacy: the emphasis was on the surgically treated subgroup and on endpoints directly relevant to hepatobiliary decision-making, including timing of surgery, operative complexity, postoperative morbidity, liver-specific postoperative risk, pathological response, margin status, recurrence, and survival. A prospectively registered public protocol was not available for the original review process; this is now stated explicitly and retained as a limitation. This systematic review was registered on the Open Science Framework (OSF) as an associated project and is available at https://osf.io/y23jt (accessed on 14 April 2026).

### 2.1. Information Sources, Search Strategy, and Reproducibility

A structured search of PubMed/MEDLINE was performed and last updated on 3 February 2026, with backward reference-list screening of included articles and adjacent reviews used as a supplementary safeguard. The search remained centered on PubMed because the review was deliberately framed as a focused synthesis of PubMed-indexed postoperative evidence; this restriction is now made explicit and treated as a methodological limitation rather than an implicit claim of exhaustive cross-database capture. The final electronic strategy was: ((“carcinoma, hepatocellular”[MeSH Terms] OR “hepatocellular carcinoma”[Title/Abstract] OR HCC[Title/Abstract]) AND (“conversion therap*”[Title/Abstract] OR downstag*[Title/Abstract] OR “conversion surg*”[Title/Abstract] OR “salvage surg*”[Title/Abstract] OR “salvage resection”[Title/Abstract] OR hepatectom*[Title/Abstract] OR “liver resection”[Title/Abstract]) AND (“immune checkpoint inhibitor*”[Title/Abstract] OR immunotherap*[Title/Abstract] OR “anti-PD-1”[Title/Abstract] OR lenvatinib[Title/Abstract] OR “tyrosine kinase inhibitor*”[Title/Abstract] OR “transarterial chemoembolization”[Title/Abstract] OR TACE[Title/Abstract] OR “hepatic arterial infusion chemotherapy”[Title/Abstract] OR HAIC[Title/Abstract])). No study-design filter was applied at the search stage. Human studies with extractable postoperative data were retained after screening, whereas reviews, editorials, conference fragments without usable resected-cohort data, transplantation studies, and clearly duplicated institutional reports were excluded.

### 2.2. Information Source and PubMed Search Strategy

**Figure 1:**
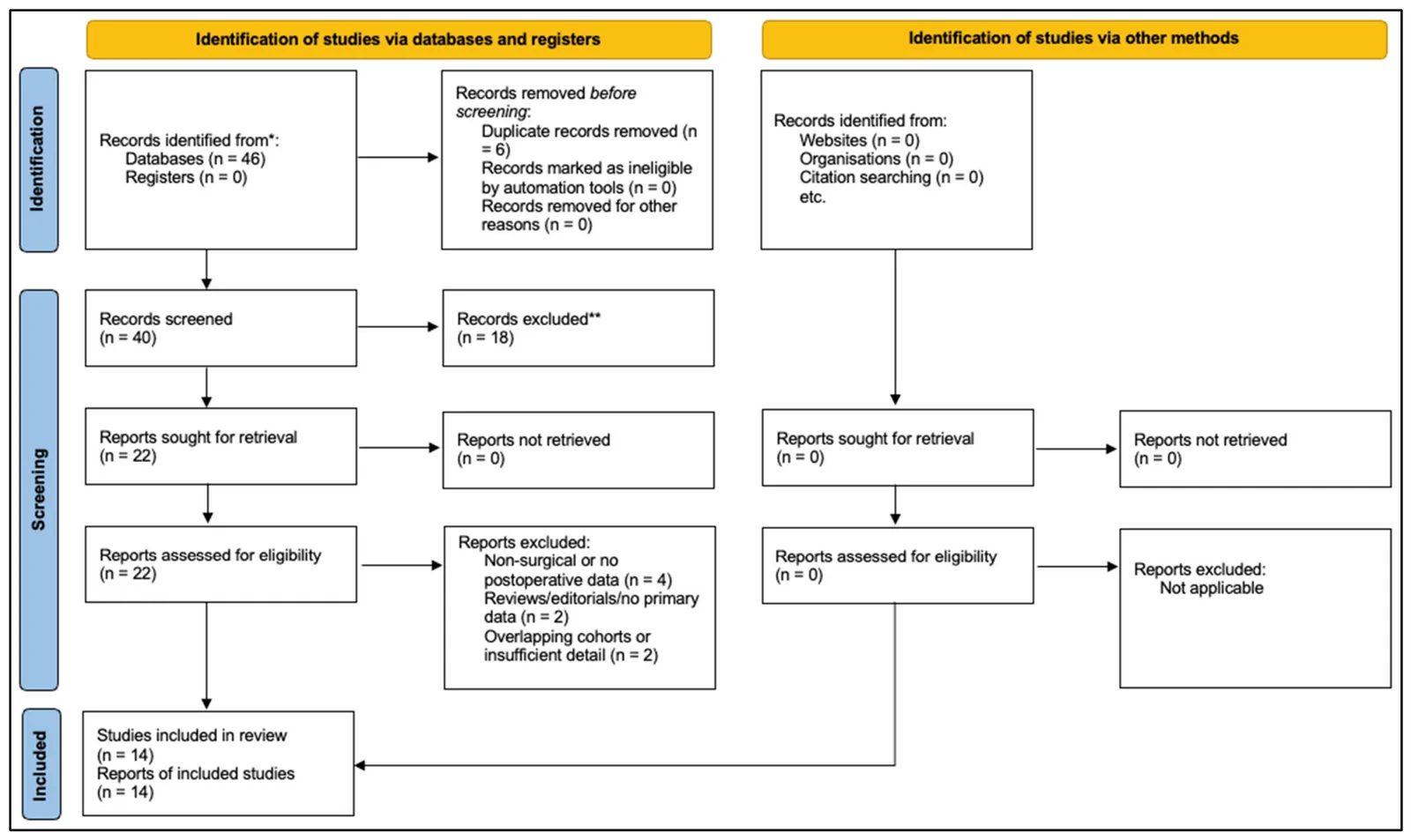
PRISMA flow diagram. * Where feasible, report the number of records identified from each database or register separately. ** If automation tools were used, report separately the number of records excluded manually and by automation.

A structured PubMed search was performed and updated through 3 February 2026. Search terms were combined iteratively to balance sensitivity and specificity, including controlled and free-text concepts related to “hepatocellular carcinoma”, “HCC”, “conversion therapy”, “downstaging”, “salvage surgery”, “conversion surgery”, “hepatectomy”, “liver resection”, “immune checkpoint inhibitor”, “anti-PD-1”, “lenvatinib”, “tyrosine kinase inhibitor”, “TACE”, and “hepatic arterial infusion chemotherapy”. Reference lists of key papers and adjacent reviews were also inspected to avoid missing large resection cohorts that might use slightly different terminology, such as “salvage resection” or “hepatectomy after conversion therapy”. The search strategy was intentionally targeted rather than excessively broad because the operative question is highly specific and many generic HCC surgery queries retrieve large volumes of irrelevant transplantation or primary resection literature. During screening, duplicate citations, overlapping early pilot reports, non-surgical cohorts, narrative reviews, conference fragments without full extractable data, and papers lacking postoperative endpoints were removed. The final search-and-screen workflow is summarized in the PRISMA diagram presented in Figure 1. Search execution details were documented contemporaneously. The PRISMA flow diagram summarizes a focused and stepwise study-selection process. A total of 46 records were initially identified, of which 6 duplicates were removed, leaving 40 citations for title and abstract screening. After this stage, 22 full-text articles were assessed for eligibility, and 14 studies were ultimately included in the final qualitative synthesis. The revised manuscript now reports the search logic explicitly and interprets these counts as those of a focused PubMed-indexed review rather than a full multi-database search.

Studies were considered eligible if they met all of the following conditions: (1) the population included patients with initially unresectable hepatocellular carcinoma; (2) the intervention involved contemporary conversion or downstaging therapy incorporating systemic treatment with or without locoregional therapy; (3) the study reported patients who subsequently underwent hepatectomy or salvage liver resection; and (4) at least one extractable postoperative endpoint was available, such as operative variables, morbidity, pathological response, recurrence-free survival, or overall survival. Both prospective and retrospective cohort designs were eligible, and comparator studies were accepted when one arm consisted of patients proceeding to surgery after conversion therapy. Reviews, editorials, letters without primary data, transplantation-focused studies, conference fragments without extractable surgical outcomes, and obviously duplicated institutional reports were excluded. Title/abstract screening, full-text eligibility confirmation, and endpoint verification were checked by two reviewers, with disagreements resolved by consensus discussion with the senior surgical author. When multiple publications appeared to derive from related high-volume experiences, reports were cross-compared by setting, publication year, regimen platform, sample size, recruitment window when available, and endpoint focus; the larger or more clinically comprehensive cohort was prioritized for overall synthesis, whereas derivative pathology or prognostic studies were retained only for non-duplicative endpoint domains.

### 2.3. Eligibility Criteria and Study Selection

For each eligible study, data were extracted into structured fields covering four layers: (i) study architecture and treatment platform; (ii) baseline external-validity domains, including the reported driver of unresectability, liver-function reserve, macrovascular invasion or PVTT, tumor burden, and extrahepatic disease or performance status when stated; (iii) perioperative and pathological outcomes, including interval to surgery, operative time, blood loss, transfusion, extent of resection, postoperative complications, post-hepatectomy liver failure, perioperative mortality, R0 status, and pathological response; and (iv) oncologic follow-up and recurrent prognostic factors. Because reported denominators varied across all treated, evaluable, resected, or pathology-assessable populations, original author-reported denominators were preserved rather than retrospectively harmonized. Missing or unclear variables were coded as not reported (NR) rather than imputed. The revised tables were specifically reorganized to separate platform heterogeneity from postoperative outcomes and to show where clinically relevant baseline or perioperative variables remained underreported.

### 2.4. Data Extraction and Outcome Domains

Formal quantitative pooling was not undertaken because heterogeneity was substantial across study design, treatment platform, definitions of unresectability, outcome denominators, and follow-up metrics. The revised manuscript therefore supplements narrative synthesis with a structured study-level appraisal based on five domains: cohort selection and resected-cohort representativeness; clarity of unresectability and eligibility definitions; control of confounding or comparator adequacy; completeness of perioperative and pathological reporting; and risk of partial cohort overlap. Across these domains, most studies were judged to have moderate or high overall concerns because surgery was offered after non-random response-based selection, comparator adjustment was limited or absent, liver-specific perioperative endpoints were inconsistently reported, and several pathology- or prognosis-focused reports may have been derived from broader institutional salvage-surgery experiences. Overlap sensitivity was handled conservatively by avoiding pooled estimates across apparently related cohorts and by interpreting derivative pathology series separately from more comprehensive operative cohorts.

### 2.5. Quality Appraisal, Heterogeneity Assessment, and Analytical Approach

The evidence base is composed of 14 studies, predominantly retrospective cohorts from China, with only one prospective phase II trial included. Table 1 summarizes the individual studies, while Table 2 stratifies the evidence by treatment platform and Table 3 maps the main external-validity domains that were or were not consistently reported. This stratified presentation clarifies that the literature does not represent a single conversion-hepatectomy population: some studies evaluated relatively discrete systemic doublets, others HAIC-based strategies, and several mixed triplet or multimodal regimens. Likewise, unresectability was variably driven by tumor burden, macrovascular invasion, liver-functional considerations, or composite multidisciplinary judgment, and these domains were not uniformly captured across reports. The size of the surgery-oriented cohorts varied markedly, from 14 to 343 patients, reflecting important heterogeneity in study scale, maturity, and susceptibility to center-specific selection effects.

## 3. Results

Table 1 shows that the evidence base is composed of 14 studies, predominantly retrospective cohorts from China, with only one prospective phase II trial included. The size of the surgery-oriented cohorts varied markedly, from as few as 14 resected patients to as many as 343, reflecting important heterogeneity in study scale and maturity. Most treatment platforms were multimodal and centered on tyrosine kinase inhibitors and anti-PD-1 therapy, often combined with TACE or HAIC, indicating that conversion surgery is currently being explored mainly in the context of intensified combination downstaging (Table 2 and Table 3).

**Figure 2:**
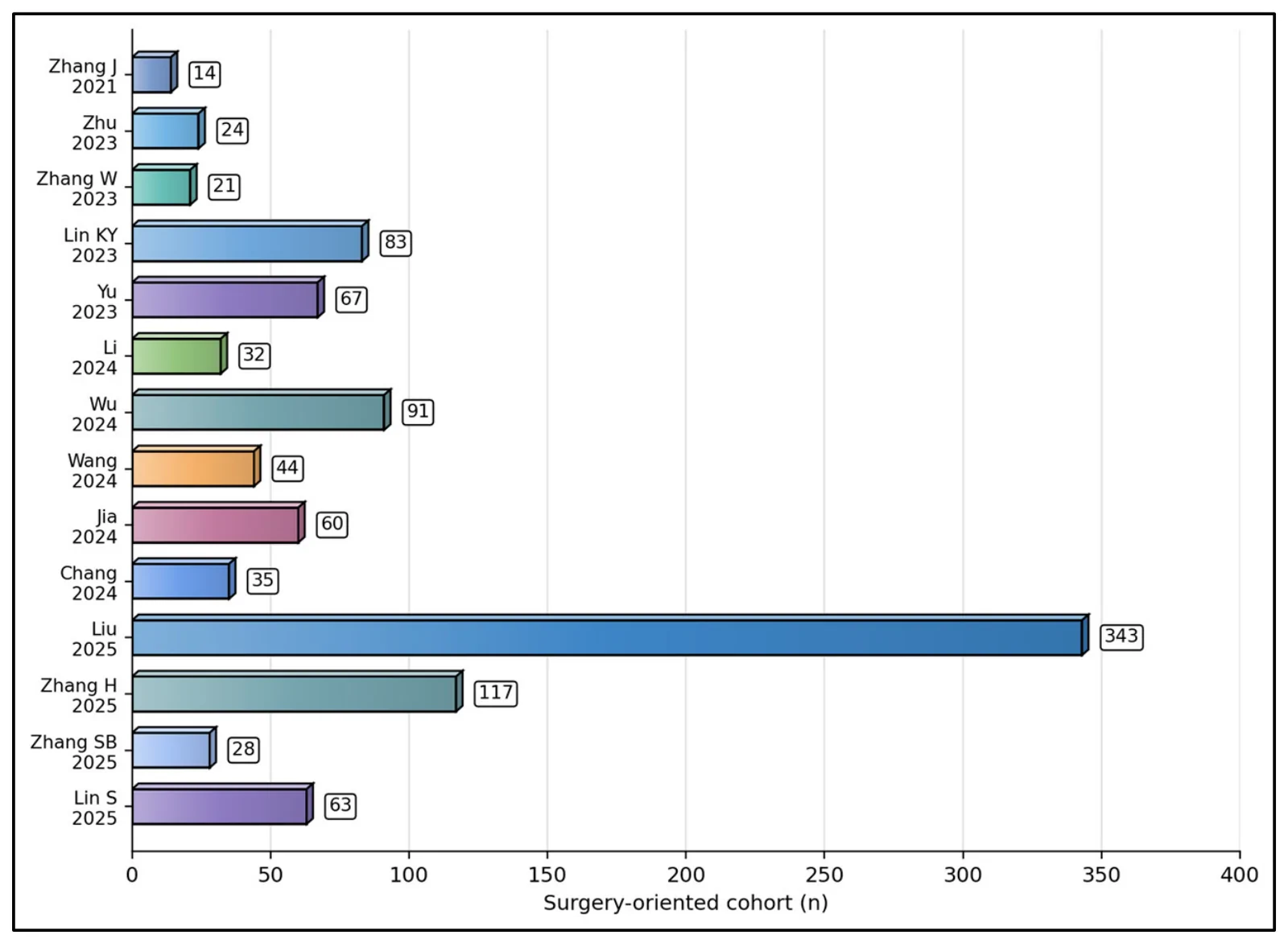
Size of the surgery-oriented cohort across the 14 included studies. Numbers adjacent to the bars indicate the author-reported cohort size used for the postoperative analysis in each publication. Data are derived from the 14 included studies [18,19,20,21,22,23,24,25,26,27,28,29,30,31].

Figure 2 illustrates the wide variation in the size of the postoperative cohorts analyzed across the included studies. Most series were relatively small to moderate, commonly including around 20 to 90 resected patients, while a few larger studies substantially influenced the overall evidence landscape, particularly the 117-patient and 343-patient cohorts.

Table 4 indicates that conversion hepatectomy can achieve substantial pathological response and high-quality resection in selected patients, but perioperative interpretation must remain cautious. Among studies reporting pathological complete response quantitatively, pCR ranged from 28.0% to 50.0%, while R0 resection reached 85.7% to 100% where stated. However, clinically decisive surgical variables were incompletely reported: extent of hepatectomy, future liver remnant strategy, bilirubin- or albumin-based reserve, post-hepatectomy liver failure beyond one study, transfusion exposure, and 30- or 90-day mortality were absent or non-uniform in many series. Accordingly, the low apparent perioperative mortality and acceptable complication profile should be interpreted as signals from selected reporting cohorts rather than as stable pooled safety estimates.

**Figure 3:**
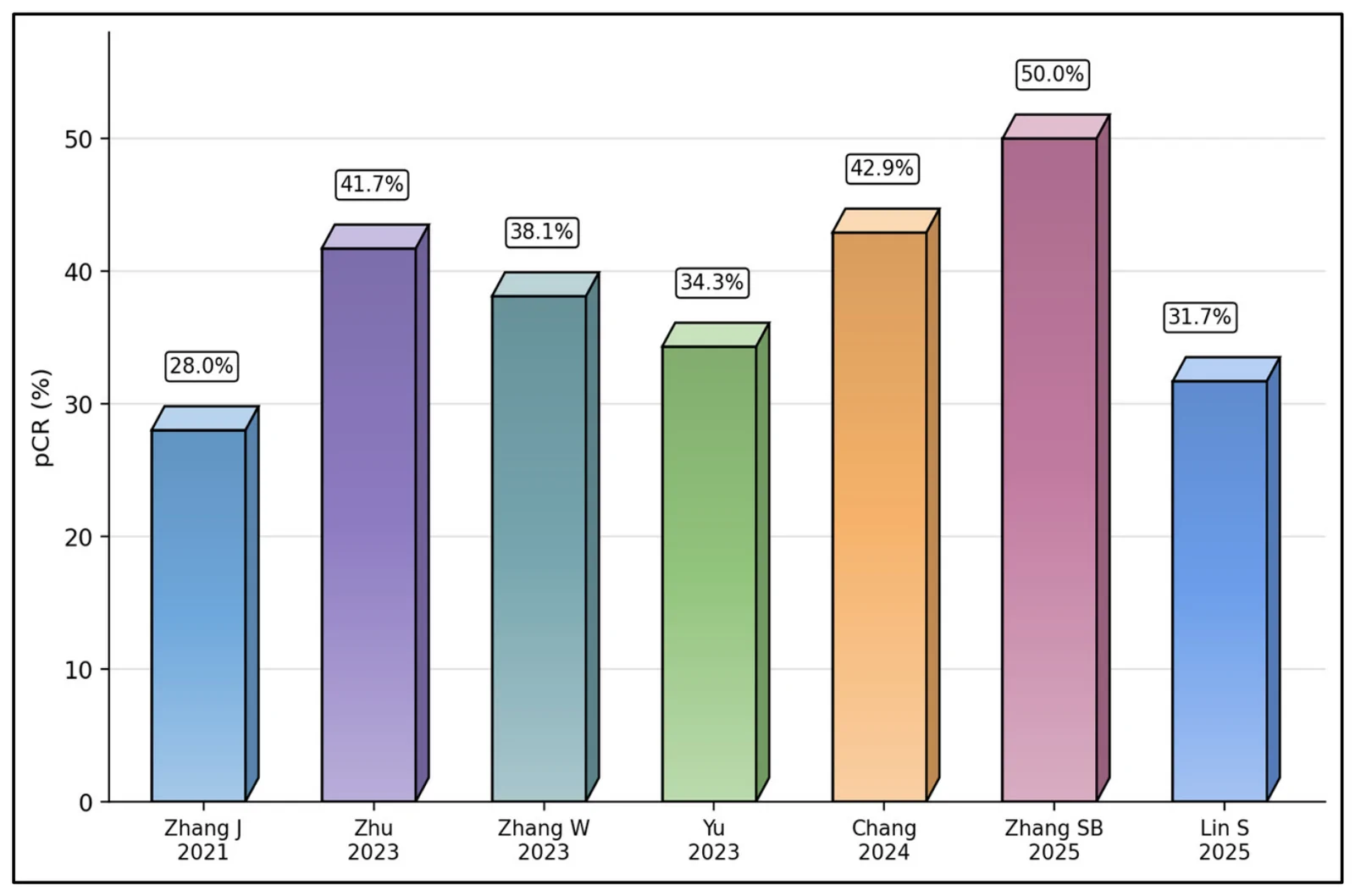
Author-reported pathological complete response (pCR) rates. Data are derived from refs [18,19,20,22,27,30,31].

Figure 3 demonstrates that deep pathological response after conversion therapy is a recurrent finding across the literature, but the denominators are not uniform and should not be pooled mechanically. In the studies that reported pathological complete response quantitatively, rates ranged from 28.0% to 50.0%, with several cohorts clustering around approximately one third to two fifths of resected or evaluable patients. This pattern suggests that modern downstaging regimens may achieve not only technical resectability but also marked biological tumor eradication in a meaningful subset of cases, while still requiring cautious interpretation because of cohort selection and reporting heterogeneity.

The first major finding of this review is that conversion hepatectomy has moved beyond anecdotal rescue surgery and is now supported by a growing, but still methodologically limited, body of postoperative evidence. The included studies consistently show that initially unresectable HCC can, in selected patients, be transformed into a resectable disease state after modern combination therapy [18,19,20,21,22,23,24,25,26,27,28,29,30,31]. This is a meaningful shift in liver-surgery practice. Historically, unresectability in HCC often implied transition to life-prolonging but non-curative treatment, whereas the current literature suggests that a subset of patients can re-enter a curative-intent pathway after response to systemic and locoregional therapy. Importantly, the studies do not support indiscriminate surgery; instead, they portray a highly selected population identified through multidisciplinary reassessment. The recurring presence of resected cohorts indicates that this is not an isolated phenomenon restricted to a single experimental center. However, the evidence also makes clear that the apparent success of conversion hepatectomy depends on institutional expertise in imaging reassessment, liver functional evaluation, technical resection planning, and longitudinal therapeutic monitoring and that the favorable results are inseparable from strong responder selection. The corresponding study-level risk-of-bias appraisal is summarised in Table 5.

The second major finding is the unexpectedly strong pathological signal. Across several resected cohorts, author-reported pCR rates clustered around one third to one half [19,20,22,27,30,31], although denominator differences must be remembered, and one additional study analyzed only patients who had already achieved pCR [26]. This matters because it provides a biological explanation for the favorable survival reported after resection in selected patients. A deep pathological response suggests that conversion therapy may be doing more than shrinking tumors radiologically; it may be altering the natural history of disease in a subset of patients sufficiently to justify surgery. The central challenge is that pathology is only visible after resection, whereas surgical decisions must be made beforehand. Consequently, clinicians need better preoperative surrogate markers of deep response. The included studies hint that AFP normalization, macrovascular response, and perhaps durable partial response may be informative [24,25,29], but the field has not yet standardized how these variables should trigger surgery.

The third major finding concerns safety and balance of benefit. Conversion hepatectomy is feasible, but the revised synthesis does not support describing it as uniformly safe or straightforward surgery. Patients often undergo resection after multiple treatment exposures, and this may affect parenchymal quality, hilar dissection, bleeding risk, and postoperative recovery. Nevertheless, the reported severe complication rates were generally within a range that experienced hepatobiliary teams would consider acceptable for major liver surgery [20,21,30]. The observation that transfusion, rather than merely resection itself, was associated with worse outcomes in one large series [21] is particularly instructive, as it implies that technical execution and perioperative management remain relevant determinants of oncologic success. Comparative studies also remind us that surgery is not automatically superior in every converted subgroup. While salvage resection was associated with better survival in some analyses [19,24,31], benefit was less obvious in certain PVTT populations and in some radiologic complete responders [25]. This nuance is clinically important and reinforces the need to individualize operative decisions.

## 4. Discussion

### 4.1. Analysis of Findings

The first major finding of this review is that conversion hepatectomy has moved beyond anecdotal rescue surgery and is now supported by a growing body of real-world postoperative evidence. The included studies consistently show that initially unresectable HCC can, in selected patients, be transformed into a resectable disease state after modern combination therapy [18,19,20,21,22,23,24,25,26,27,28,29,30,31]. This is a meaningful shift in liver-surgery practice. Historically, unresectability in HCC often implied transition to life-prolonging but non-curative treatment, whereas the current literature suggests that a subset of patients can re-enter a curative-intent pathway after response to systemic and locoregional therapy. Importantly, the studies do not support indiscriminate surgery; instead, they portray a highly selected population identified through multidisciplinary reassessment. The recurring presence of sizable resected cohorts indicates that this is not an isolated phenomenon restricted to a single experimental center. However, the evidence also makes clear that the success of conversion hepatectomy depends on institutional expertise in imaging reassessment, liver functional evaluation, technical resection planning, and longitudinal therapeutic monitoring. In practical terms, the message is neither that all responders should be resected nor that surgery should be deferred automatically once systemic control is achieved, but that resectability must now be reconsidered dynamically throughout treatment.

The second major finding is the unexpectedly strong pathological signal. Across several resected cohorts, author-reported pCR rates clustered around one third to one half [19,20,22,27,30,31], although denominator differences must be remembered, and one additional study analyzed only patients who had already achieved pCR [26]. This matters because it provides a biological explanation for the favorable survival reported after resection in selected patients. A deep pathological response suggests that conversion therapy may be doing more than shrinking tumors radiologically; it may be altering the natural history of disease in a subset of patients sufficiently to justify surgery. The central challenge is that pathology is only visible after resection, whereas surgical decisions must be made beforehand. Consequently, clinicians need better preoperative surrogate markers of deep response. The included studies hint that AFP normalization, macrovascular response, and perhaps durable partial response may be informative [24,25,29], but the field has not yet standardized how these variables should trigger surgery. Still, the available evidence strongly argues that pathological response should no longer be treated as an optional exploratory endpoint. It should be regarded as a core measure in future trials of conversion therapy because it may help explain why some patients experience long postoperative remission while others recur despite technically successful resection.

An equally important message from the revised synthesis is what remains missing. Even though the clinical question is inherently perioperative, the literature only inconsistently reports extent of resection, background liver status beyond broad Child–Pugh or ALBI orientation, portal hypertension, future liver remnant strategy, bile leakage, post-hepatectomy liver failure, and standardized 30- or 90-day mortality. This omission matters because conversion candidates are biologically and surgically different from upfront resection cohorts: they often arrive after prolonged systemic exposure, repeated TACE or HAIC, or treatment-related inflammation and fibrosis. Without granular perioperative reporting, clinicians cannot determine whether favorable survival reflects biologic responder selection alone or whether specific operative pathways and liver-reserve thresholds contribute independently to safety.

This review has several limitations. First, the search strategy was intentionally focused on PubMed/MEDLINE with backward citation chaining rather than a fully exhaustive multi-database capture, so relevant studies indexed only in Embase, Web of Science, Scopus, CENTRAL, or trial registries may have been missed. Second, a prospectively registered public protocol was not available. Third, the evidence base is dominated by retrospective East Asian cohorts with strong response-based selection for surgery, limiting generalizability. Fourth, definitions of unresectability, conversion success, pathological response, recurrence endpoints, and reporting denominators were inconsistent across studies. Fifth, perioperative variables most relevant to hepatobiliary surgeons—extent of resection, liver reserve, future liver remnant management, post-hepatectomy liver failure, transfusion, and perioperative mortality—were incompletely reported. Sixth, some cohorts may be partially overlapping at the institutional or temporal level despite conservative handling. These constraints justify a cautious, hypothesis-generating interpretation.

The third major finding concerns safety and balance of benefit. Conversion hepatectomy is feasible, but the review does not support calling it easy surgery. Patients often undergo resection after multiple treatment exposures, and this may affect parenchymal quality, hilar dissection, bleeding risk, and postoperative recovery. Nevertheless, the reported severe complication rates were generally within a range that experienced hepatobiliary teams would consider acceptable for major liver surgery [20,21,30]. The observation that transfusion, rather than merely resection itself, was associated with worse outcomes in one large series [21] is particularly instructive, as it implies that technical execution and perioperative management remain relevant determinants of oncologic success. Comparative studies also remind us that surgery is not automatically superior in every converted subgroup. While salvage resection was associated with better survival in some analyses [19,24,31], benefit was less obvious in certain PVTT populations and in some radiologic complete responders [25]. This nuance is clinically important. Modern HCC surgery after conversion therapy should not be framed as a binary race to the operating room but as a selective intervention whose value depends on the interaction between response depth, disease pattern, liver reserve, and residual risk of occult dissemination.

### 4.2. Study Limitations

This review has several limitations. First, the available evidence is dominated by retrospective observational studies from East Asia, limiting global external validity. Second, definitions of unresectability, conversion success, pathological response, and survival endpoints were inconsistent across studies, reducing comparability. Third, some cohorts may be partially overlapping at the institutional or temporal level despite careful screening, especially where later publications examined specific pathological or prognostic subgroups derived from broader salvage-surgery experience. Fourth, many reports provided incomplete perioperative detail, preventing formal pooled analysis of morbidity or liver-failure risk.

## 5. Conclusions

In conclusion, the contemporary literature suggests that conversion hepatectomy may be a credible curative-intent option for highly selected patients with initially unresectable HCC who respond to modern combination downstaging and are reassessed in experienced multidisciplinary centers. The most reproducible positive signals are high R0 resection rates, frequent deep pathological response, and encouraging early survival among patients who actually reach surgery. However, the current evidence base is observational, heterogeneous, regionally concentrated, incompletely reported, and potentially affected by cohort overlap. Conversion hepatectomy should therefore be viewed as an emerging, carefully selected strategy rather than an already standardized pathway, and future multicenter prospective registries should harmonize unresectability definitions, baseline liver-function reporting, perioperative endpoints, pathological-response metrics, and overlap-free longitudinal follow-up.

## Figures and Tables

**Table 1:** Characteristics of included studies, treatment platforms, and surgery-oriented cohorts.

Main Analytical Focus	Surgery-Oriented Cohort	Total Treated Cohort	Dominant Conversion Regimen	Design/Setting	Study
Conversion feasibility and pathological response	14 resected	34 enrolled; 25 evaluable	Angiogenesis inhibitor + anti-PD-1 antibody + HAIC	Retrospective single-center China	[18] Zhang J et al., 2021
Predictors of conversion resection and postoperative survival	24 resected	101 treated	TKI + anti-PD-1 antibody	Retrospective single-center China	[19] Zhu et al., 2023
Prospective conversion efficacy, pathology, and surgical safety	21 resected	56 enrolled	Lenvatinib + anti-PD-1 antibody	Phase II single-arm trial China	[20] Zhang W et al., 2023
Perioperative safety and oncologic outcomes of salvage resection	83 resected	Surgical cohort only (83)	TACE + TKI + anti-PD-1 antibody	Retrospective multicenter China	[21] Lin KY et al., 2023
Pathology and prognosis after hepatectomy	67 resected	Surgical cohort only (67)	HAIC + TKI + anti-PD-1 antibody	Retrospective single-center China	[22] Yu et al., 2023
Comparison between conversion surgery and primary surgery cohorts	32 resected after conversion	32 conversion-surgery vs. 419 upfront-surgery comparator	Lenvatinib + TACE + PD-1 inhibitor	Real-world observational with comparator China	[23] Li et al., 2024
Salvage surgery versus non-surgery after conversion therapy	91 salvage surgery/53 non-surgery	144 assessed	TACE + lenvatinib + anti-PD-1 antibody	Retrospective multicenter comparator study China	[24] Wu et al., 2024
Role of surgery in PVTT after conversion	44 surgery/49 no surgery	93 converted PVTT cases	Locoregional therapy + TKI + anti-PD-1 antibody in PVTT	Retrospective comparator study China	[25] Wang et al., 2024
Outcomes after pathological complete response	60 resected with pCR	pCR-selected surgical cohort (60)	Mixed conversion regimens leading to pCR	Retrospective pathology-focused study China	[26] Jia et al., 2024
Single-center summary of evolving conversion practice	35 radical resections	38 successfully converted	Multiple conversion regimens over four years	Retrospective single-center consecutive cohort China	[27] Chang et al., 2024
Predictors of recurrence and death after conversion hepatectomy	343 resected	Surgical cohort only (343)	Hepatectomy after conversion therapy	Large retrospective prognostic cohort China	[28] Liu et al., 2025
Tumor regression grade and recurrence after surgery	117 resected	Surgical cohort only (117)	Mixed conversion regimens; many received TACE + targeted + immunotherapy	Retrospective pathology-prognosis study China	[29] Zhang H et al., 2025
Efficacy and safety of salvage surgery	28 resected	117 enrolled	Mixed conversion regimens after triplet therapy	Retrospective salvage-surgery cohort China	[30] Zhang SB et al., 2025
Treatment platform and survival impact of conversion resection	63 resected	301 included	TACE/HAIC-based combination treatment	Retrospective cohort with comparator China	[31] Lin S et al., 2025

**Table 2:** Stratified presentation of included studies by conversion-treatment platform.

Treatment Platform Stratum	Included Studies	No. of Studies	Surgery-Oriented Patients	Main Interpretive Issue
High ORR (96.0%) enabled resection in selected patients	NR	NR	NR	[18] Zhang J et al., 2021
Hepatectomy independently favored OS (HR 0.050, 95% CI 0.007–0.365); pCR trended toward better RFS	12-mo OS 95.8%	12-mo RFS 75.0%	Median 21.5 mo	[19] Zhu et al., 2023
Prospective conversion feasibility shown; higher pretreatment CD8+ enrichment associated with response	Median OS 23.9 mo in treated cohort	12-mo RFS 47.6% among resected patients	Median 23.5 mo in overall cohort	[20] Zhang W et al., 2023
pCR and transfusion were independent RFS determinants	1-/2-y OS 92.2%/87.3%	Median RFS 25.4 mo; 1-/2-y RFS 68.2%/61.8%	Median 15.1 mo	[21] Lin KY et al., 2023
pCR and absence of MVI associated with better prognosis; tumor number associated with pCR	Median OS 28.7 mo	Median DFS/RFS 19.3 mo	Median 15.9 mo	[22] Yu et al., 2023
Conversion surgery associated with improved EFS (HR 0.231, 95% CI 0.105–0.504)	OS similar after matching	EFS not reached vs. 12.9 mo before matching; improved after matching	NR	[23] Li et al., 2024
Overall OS favored salvage surgery	1-/2-y OS 92.0%/79.9% vs. 85.5%/39.6% (surgery vs. non-surgery)	No significant overall PFS difference	NR	[24] Wu et al., 2024
Baseline AFP and best mRECIST response predicted OS/PFS; CR or types III–IV PVTT trended toward no-surgery benefit	No overall OS advantage (*p* = 0.370)	No overall PFS advantage (*p* = 0.334)	NR	[25] Wang et al., 2024
Excellent outcomes in pCR-selected cohort; no significant adjuvant-therapy RFS signal	1-/3-y OS 98.3%/95.6%	1-/3-y RFS 81.1%/71.4%	NR	[26] Jia et al., 2024
Only 1.81% of unresectable HCC patients were converted to radical resection in this single-center experience	OS NR in abstract	1-y DFS 86.8% after conversion surgery	Median 19.3 mo	[27] Chang et al., 2024
Tumor number, AFP response, tumor response, and successful downstaging predicted RFS; ALBI and AFP response predicted OS	OS predictors reported; median OS NR in abstract	157/343 (45.8%) recurred or metastasized	Median time to recurrence 16.7 mo among recurrent cases	[28] Liu et al., 2025
TRG2 HR 4.25 and TRG3 HR 6.20 for recurrence; rapid AFP normalization favored optimal TRG	NR	Median RFS not reached in TRG1a/1b; 16.7 mo in TRG2 and 14.6 mo in TRG3	Median 19.3 mo	[29] Zhang H et al., 2025
pCR and preoperative albumin predicted RFS	1-/2-y OS 92.7%/87.6%	1-/2-y RFS 75.0%/59.4%	Median 15.0 mo	[30] Zhang SB et al., 2025
Radiographic complete response and surgery were associated with better outcomes	OS not reached vs. 58.5 mo	Median PFS 42.83 vs. 9.7 mo (surgery vs. non-surgery)	NR	[31] Lin S et al., 2025
Systemic doublet (TKI + anti-PD-1/lenvatinib + anti-PD-1)	[19,20]	2	45	More homogeneous platform, but still small single-center cohorts.
Systemic + HAIC strategies	[18,22]	2	81	Strong biological response signal, but limited perioperative detail.
Locoregional–systemic triplet or multimodal conversion platforms	[21,23,24,25,30,31]	6	341	Largest clinically relevant subgroup, but marked denominator and comparator heterogeneity.
Mixed evolving regimens or pathology-focused post-conversion cohorts	[26,27,28,29]	4	555	Useful for prognosis/pathology, but vulnerable to selection enrichment and overlap concerns.

**Table 3:** External-validity domains and reporting consistency across the included studies.

Domain	Reporting Pattern Across Studies	Observed Trend	Implication
Driver of unresectability	Variably described	Tumor burden, PVTT, liver reserve, or composite multidisciplinary judgment were used non-uniformly.	Limits direct comparison of who was considered convertible to surgery.
Baseline liver function	Incompletely reported	Child–Pugh/ALBI orientation was often implied, but detailed reserve metrics were not consistently extractable.	Weakens external validity for perioperative applicability.
Macrovascular invasion/PVTT	Explicit in only a subset	One cohort focused on PVTT, whereas others mixed vascular-invasion status.	Makes survival and pCR comparisons vulnerable to case-mix imbalance.
Tumor burden/extrahepatic disease/performance status	Non-uniform	Some cohorts emphasized locally advanced intrahepatic disease, whereas others did not map these variables clearly.	Restricts cross-study interpretation of oncologic risk.
Perioperative detail	Often incomplete	Extent of resection, FLR strategy, transfusion, PHLF, and perioperative mortality were inconsistently reported.	Prevents robust surgical benchmarking.

**Table 4:** Perioperative and pathological outcomes after conversion hepatectomy.

Pathological Response	R0	Postoperative Morbidity/Periop. Death	Blood Loss	Op. Time	Interval to Surgery	Study
pCR 28.0% of evaluable cohort (7/25); 60.0% surgical conversion rate	NR	NR	NR	NR	Median time to response 50.5 d; surgery interval NR	[18] Zhang J et al., 2021
pCR 41.7% (10/24)	R0 100% (24/24)	Detailed graded morbidity NR in abstract	NR	NR	Median 3.9 mo from systemic therapy to resection	[19] Zhu et al., 2023
pCR 38.1% (8/21); pPR 42.9%	R0 85.7%	Postop complications 14.3%; grade III 9.5%; PHLF-A 19.4%; 0 perioperative deaths reported	NR	NR	Median 109 d to surgery	[20] Zhang W et al., 2023
pCR reported as prognostic variable; exact overall rate NR	NR	Overall 48.2%; major 16.9%; 1 perioperative death	Median 400 mL	Median 200 min	NR	[21] Lin KY et al., 2023
pCR 34.3% (23/67)	NR	NR	NR	NR	Median 4.0 mo to surgery	[22] Yu et al., 2023
Lower MVI after conversion (3.1% vs. 50.4% in upfront surgery)	NR	Similar safety vs. upfront surgery	NR	NR	NR	[23] Li et al., 2024
NR	NR	Postoperative morbidity not separately reported for surgery arm	NR	NR	NR	[24] Wu et al., 2024
NR	NR	Conversion-treatment adverse events comparable between surgery and non-surgery groups; postoperative morbidity not separately reported	NR	NR	NR	[25] Wang et al., 2024
All included patients had pCR by design	NR	NR	NR	NR	NR	[26] Jia et al., 2024
Author-reported pCR 42.9%	35 radical resections	Severe TRAEs 44.7% during conversion; postoperative morbidity NR	NR	NR	NR	[27] Chang et al., 2024
NR	NR	NR	NR	NR	NR	[28] Liu et al., 2025
TRG1a 29, TRG1b 13, TRG2 23, TRG3 52	NR	NR	NR	NR	Operation interval varied by TRG; longest in TRG1a (median 23.7 weeks)	[29] Zhang H et al., 2025
pCR 50.0% (14/28)	NR	Overall postop 71.4%; Clavien–Dindo III–V 14.3%	NR	NR	NR	[30] Zhang SB et al., 2025
pCR 31.7% (20/63)	R0 100%	NR	NR	NR	NR	[31] Lin S et al., 2025

**Table 5:** Risk of bias assessment.

Study	Design	Selection/Confounding Concern	Reporting Concern	Potential Overlap Concern	Overall Concern
[18] Zhang J et al., 2021	Retrospective single-center	High response-based surgical selection; no controlled comparator	Limited perioperative detail	Low	High
[19] Zhu et al., 2023	Retrospective single-center	Selected resected responders; modest cohort size	Moderate	Low	Moderate–high
[20] Zhang W et al., 2023	Phase II single-arm	Prospective enrollment but no randomized comparator	Moderate	Low	Moderate
[21] Lin KY et al., 2023	Retrospective multicenter	Comparator/confounding control limited	Better perioperative detail but incomplete denominator harmonization	Moderate	Moderate
[22] Yu et al., 2023	Retrospective single-center	Selected operative cohort	Limited perioperative reporting	Moderate	Moderate–high
[23] Li et al., 2024	Observational comparator	Comparator available, but non-random baseline imbalance remains	Moderate	Low	Moderate
[24] Wu et al., 2024	Retrospective multicenter comparator	Surgery versus non-surgery strongly vulnerable to treatment-selection bias	Postoperative data incompletely separated	Moderate	High
[25] Wang et al., 2024	Retrospective comparator in PVTT	Highly selected disease subset	Postoperative outcomes not fully granular	Low–moderate	Moderate–high
[26] Jia et al., 2024	Retrospective pCR-selected cohort	Extreme enrichment for favorable responders	Not designed for general perioperative benchmarking	Moderate	High
[27] Chang et al., 2024	Retrospective single-center	Evolving practice cohort	Postoperative details limited	Moderate	Moderate–high
[28] Liu et al., 2025	Large retrospective prognostic cohort	No non-surgical control; selected resected population	Better prognosis detail than perioperative detail	Moderate	Moderate–high
[29] Zhang H et al., 2025	Retrospective pathology-prognosis cohort	Selected resected subset	Strong pathology detail; perioperative detail sparse	Moderate	Moderate–high
[30] Zhang SB et al., 2025	Retrospective salvage cohort	No randomized comparator	Complication reporting present but limited baseline mapping	Low	Moderate–high
[31] Lin S et al., 2025	Retrospective comparator cohort	Comparator present but residual confounding likely	Perioperative detail sparse	Moderate	Moderate–high

## Data Availability

No new data were created or analyzed in this study. Data sharing is not applicable to this article.

## Supplementary Materials

The following supporting information can be downloaded at: https://www.mdpi.com/article/10.3390/curroncol33080453/s1, Table S1: PRISMA_2020_checklist.

## References

[1] Singal A.G., Lampertico P., Nahon P. (2020). Epidemiology and surveillance for hepatocellular carcinoma: New trends. J. Hepatol..

[2] Llovet J.M., Kelley R.K., Villanueva A., Singal A.G., Pikarsky E., Roayaie S., Lencioni R., Koike K., Zucman-Rossi J., Finn R.S. (2021). Hepatocellular carcinoma. Nat. Rev. Dis. Prim..

[3] Brown Z.J., Tsilimigras D.I., Ruff S.M., Mohseni A., Kamel I.R., Cloyd J.M., Pawlik T.M. (2023). Management of hepatocellular carcinoma: A review. JAMA Surg..

[4] Marrero J.A., Kulik L.M., Sirlin C.B., Zhu A.X., Finn R.S., Abecassis M.M., Roberts L.R., Heimbach J.K. (2018). Diagnosis, staging, and management of hepatocellular carcinoma: 2018 practice guidance by the American Association for the Study of Liver Diseases. Hepatology.

[5] Singal A.G., Llovet J.M., Yarchoan M., Mehta N., Heimbach J.K., Dawson L.A., Jou J.H., Kulik L.M., Agopian V.G., Marrero J.A. (2023). AASLD practice guidance on prevention, diagnosis, and treatment of hepatocellular carcinoma. Hepatology.

[6] European Association for the Study of the Liver (2018). EASL Clinical Practice Guidelines: Management of hepatocellular carcinoma. J. Hepatol..

[7] Finn R.S., Qin S., Ikeda M., Galle P.R., Ducreux M., Kim T.Y., Kudo M., Breder V., Merle P., Kaseb A.O. (2020). Atezolizumab plus bevacizumab in unresectable hepatocellular carcinoma. N. Engl. J. Med..

[8] Kudo M., Finn R.S., Qin S., Han K.H., Ikeda K., Piscaglia F., Baron A., Park J.W., Han G., Jassem J. (2018). Lenvatinib versus sorafenib in first-line treatment of patients with unresectable hepatocellular carcinoma: A randomised phase 3 non-inferiority trial. Lancet.

[9] Finn R.S., Ikeda M., Zhu A.X., Sung M.W., Baron A.D., Kudo M., Okusaka T., Kobayashi M., Kumada H., Kaneko S. (2020). Phase Ib study of lenvatinib plus pembrolizumab in patients with unresectable hepatocellular carcinoma. J. Clin. Oncol..

[10] Llovet J.M., Montal R., Sia D., Finn R.S. (2018). Molecular therapies and precision medicine for hepatocellular carcinoma. Nat. Rev. Clin. Oncol..

[11] Sun H.C., Zhou J., Wang Z., Liu X., Xie D.Y., Xu J., Ren Z., Fan J., Tang Z., Zhou J. (2022). Chinese expert consensus on conversion therapy for hepatocellular carcinoma (2021 edition). Hepatobiliary Surg. Nutr..

[12] Song T., Lang M., Ren S., Gan L., Lu W. (2021). The past, present and future of conversion therapy for liver cancer. Am. J. Cancer Res..

[13] Arita J., Ichida A., Nagata R., Mihara Y., Kawaguchi Y., Ishizawa T., Akamatsu N., Kaneko J., Hasegawa K. (2022). Conversion surgery after preoperative therapy for advanced hepatocellular carcinoma in the era of molecular targeted therapy and immune checkpoint inhibitors. J. Hepatobiliary Pancreat. Sci..

[14] Vogel A., Meyer T., Sapisochin G., Salem R., Saborowski A. (2022). Hepatocellular carcinoma. Lancet.

[15] Llovet J.M., Pinyol R., Yarchoan M., Singal A.G., Marron T.U., Schwartz M., Pikarsky E., Kudo M., Finn R.S. (2024). Adjuvant and neoadjuvant immunotherapies in hepatocellular carcinoma. Nat. Rev. Clin. Oncol..

[16] Page M.J., McKenzie J.E., Bossuyt P.M., Boutron I., Hoffmann T.C., Mulrow C.D., Shamseer L., Tetzlaff J.M., Akl E.A., Brennan S.E. (2021). The PRISMA 2020 statement: An updated guideline for reporting systematic reviews. BMJ.

[17] Campbell M., McKenzie J.E., Sowden A., Katikireddi S.V., Brennan S.E., Ellis S., Hartmann-Boyce J., Ryan R., Shepperd S., Thomas J. (2020). Synthesis without meta-analysis (SWiM) in systematic reviews: Reporting guideline. BMJ.

[18] Zhang J., Zhang X., Mu H., Yu G., Xing W., Wang L., Zhang T. (2021). Surgical conversion for initially unresectable locally advanced hepatocellular carcinoma using a triple combination of angiogenesis inhibitors, anti-PD-1 antibodies, and hepatic arterial infusion chemotherapy: A retrospective study. Front. Oncol..

[19] Zhu X.-D., Huang C., Shen Y.-H., Xu B., Ge N.-L., Ji Y., Qu X.-D., Chen L., Chen Y., Li M.-L. (2023). Hepatectomy after conversion therapy using tyrosine kinase inhibitors plus anti-PD-1 antibody therapy for patients with unresectable hepatocellular carcinoma. Ann. Surg. Oncol..

[20] Zhang W., Tong S., Hu B., Wan T., Tang H., Zhao F., Jiao T., Li J., Zhang Z., Cai J. (2023). Lenvatinib plus anti-PD-1 antibodies as conversion therapy for patients with unresectable intermediate-advanced hepatocellular carcinoma: A single-arm, phase II trial. J. Immunother. Cancer.

[21] Lin K.-Y., Lin Z.-W., Chen Q.-J., Luo L.-P., Zhang J.-X., Chen J.-H., Wang K., Tai S., Zhang Z.-B., Wang S.-F. (2023). Perioperative safety, oncologic outcome, and risk factors of salvage liver resection for initially unresectable hepatocellular carcinoma converted by transarterial chemoembolization plus tyrosine kinase inhibitor and anti-PD-1 antibody: A retrospective multicenter study of 83 patients. Hepatol. Int..

[22] Yu B., Zhang N., Feng Y., Zhang Y., Zhang T., Wang L. (2023). Hepatectomy after conversion therapy with hepatic arterial infusion chemotherapy, tyrosine kinase inhibitors and anti-PD-1 antibodies for initially unresectable hepatocellular carcinoma. J. Hepatocell. Carcinoma.

[23] Li X., Wang X., Bai T., Chen J., Lu S., Wei T., Tang Z., Zhao G., Lu H., Li L. (2024). Conversion surgery for initially unresectable hepatocellular carcinoma using lenvatinib combined with TACE plus PD-1 inhibitor: A real-world observational study. Dig. Liver Dis..

[24] Wu J.-Y., Fu Y.-K., Ou X.-Y., Li S.-Q., Zhang Z.-B., Zhou J.-Y., Li B., Wang S.-J., Chen Y.-F., Yan M.-L. (2024). Outcomes of salvage surgery versus non-salvage surgery for initially unresectable hepatocellular carcinoma after conversion therapy with transcatheter arterial chemoembolization combined with lenvatinib plus anti-PD-1 antibody: A multicenter retrospective study. Ann. Surg. Oncol..

[25] Wang L., Feng J.-K., Lu C.-D., Wu J.-Y., Zhou B., Wang K., Wei X.-B., Liang C., Zhou H.-K., Shi J. (2024). Salvage surgery for initially unresectable HCC with PVTT converted by locoregional treatment plus tyrosine kinase inhibitor and anti-PD-1 antibody. Oncologist.

[26] Jia J., Ding C., Mao M., Gao F., Shao Z., Zhang M., Zheng S. (2024). Pathological complete response after conversion therapy in unresectable hepatocellular carcinoma: A retrospective study. BMC Gastroenterol..

[27] Chang Z., Li M., Sun Z., Liu Z., Yang Y., Xu L., Li L., Zhang C., Sun P., Zhong J. (2024). Clinical study on conversion therapy of hepatocellular carcinoma: Summary and comparison of clinical data from a single center of consecutive four years. BMC Gastroenterol..

[28] Liu S., Wu Z., Wang C., Qiao L., Huang Z., Yuan Y., Zou R., He W., Li B., Yuan Y. (2025). Prognosis predictors of hepatocellular carcinoma after hepatectomy following conversion therapy. Eur. J. Surg. Oncol..

[29] Zhang H., Yang T., Huang L., Li B., Xu H., Wei Y. (2025). Tumor regression grade predicts recurrence-free survival in intermediate-advanced hepatocellular carcinoma after conversion therapy. Front. Immunol..

[30] Zhang S.B., Nashan B., Wang Y.L., Zhang S.G. (2025). Efficacy of salvage surgery for hepatocellular carcinoma following conversion therapy. World J. Clin. Oncol..

[31] Lin S., Song Z., Chen P., Yu X., Xie W., Hua Y., Li S., Shen S., Kuang M. (2025). Conversion Therapy Based on TACE/HAIC-Based Treatment to Improve the Therapeutic Effect of Initially Unresectable Hepatocellular Carcinoma. Liver Cancer.

